# Maternal Bivalent Respiratory Syncytial Virus Prefusion F (RSVpreF) Vaccination in Panama: A Retrospective Follow-Up Study

**DOI:** 10.7759/cureus.116644

**Published:** 2026-09-21

**Authors:** Ana Lucas Quintero, Rodrigo DeAntonio, Isaac Fernandez Camargo, Ana Peralta Alvarado, Luis Escobar, Belen Ricci Perez, Luis Alberto Flores, Cynthia Gadea, Sofia Barrios, Emmanuel Guillen, Abigail De Avila, Jaime Ricardo Sevillano Giraldez

**Affiliations:** 1 Pediatrics, Vacunar Panama, Panama, PAN; 2 Department of Population Intelligence and Scientific Advancement, Endpoints Research Sites Network &amp; CEVAXIN, Panama, PAN; 3 Faculty of Medicine, Universidad de Panamá, Panama, PAN; 4 Pediatrics, Pediatrics Clinic Panama, Panama, PAN; 5 Population Intelligence and Biostatistics, Cevaxin, Panama, PAN; 6 Faculty of Medicine, School of Public Health, Universidad de Panamá, Panama, PAN

**Keywords:** maternal vaccination, panama, pharmacovigilance, pregnancy, respiratory syncytial virus, rsvpref, vaccine safety

## Abstract

Background: Panama introduced routine maternal bivalent respiratory syncytial virus prefusion F (RSVpreF) vaccination (Abrysvo, Pfizer Inc., New York, NY) in July 2025. We described common retrospectively self-reported reactions and maternal, perinatal, and neonatal conditions among the first 200 eligible recipients enrolled in active follow-up.

Methods: This retrospective descriptive cohort included women aged 18 years or older who received RSVpreF at 32.0-36.6 weeks of gestation from July through December 2025 and completed one structured interview, generally after delivery, from January through July 2026. Exact vaccination-to-interview intervals were unavailable. Frequencies and Wilson 95% confidence intervals were calculated.

Results: Of 225 completed questionnaires, 200 met the eligibility criteria. The mean maternal age was 28.1 years (standard deviation = 6.4). Any reaction was reported by 55.5% (95% CI: 48.6%-62.2%), most often injection-site pain (48.0%), and self-reported fever occurred in 2.5%. A participant-reported hypertensive disorder occurred in 12.0%. Four births were preterm (2.0%), and five newborns had low birth weight (2.5%). Participant-reported jaundice was the most frequent neonatal condition (20.0%).

Conclusions: Reported reactions were mainly local and short-lived. These findings describe early implementation in Panama but do not assess vaccine effectiveness, comparative safety, rare-event risk, or causality. Convenience recruitment, post-delivery selection, retrospective recall, absence of clinical verification and a comparator, and missing baseline risk and event-timing data limit interpretation.

## Introduction

Respiratory syncytial virus (RSV) is a major cause of acute lower respiratory infection and hospitalization in young children. In 2019, an estimated 33 million RSV-associated episodes and 101,400 deaths occurred among children younger than five years; infants younger than six months accounted for approximately 1.4 million hospital admissions and 45,700 deaths [1]. A prospective birth cohort in Panama and El Salvador also documented substantial RSV-associated illness during the first two years of life, with the highest incidence among infants younger than three months [2].

Panama has a tropical respiratory-virus ecology, with RSV detected throughout the year and activity generally increasing during the rainy season [3]. In July 2025, the country introduced the bivalent RSV prefusion F (RSVpreF) vaccine (Abrysvo, Pfizer Inc., New York, NY) for maternal administration at 32.0-36.6 weeks of gestation [4]. The phase 3 MATISSE trial established efficacy against severe RSV-associated lower respiratory tract illness in early infancy [5]. The World Health Organization (WHO) and Pan American Health Organization (PAHO) have emphasized that country introduction should be accompanied by coverage monitoring and maternal and neonatal safety surveillance [6,7]. Early post-introduction studies in Argentina subsequently documented effectiveness against RSV-associated hospitalization in young infants [8,9]. The present study did not evaluate vaccine effectiveness; its purpose was early post-introduction safety follow-up in Panama.

Safety evaluation remains important because MATISSE reported a numerical imbalance in preterm birth (5.7% among RSVpreF recipients and 4.7% with placebo), with heterogeneity across settings [10]. Large post-authorization studies can estimate uncommon outcomes, but timely local surveillance has a distinct role: it characterizes commonly reported reactions under routine delivery, tests follow-up systems, identifies outcomes requiring clinical verification, and informs risk communication in the population where the program is implemented.

Panama was an early Central American adopter of routine maternal RSVpreF vaccination. At study initiation, no Panamanian post-introduction data had been published. The novelty of this study is therefore primarily geographic and programmatic. We aimed to estimate the frequency of common retrospectively self-reported reactions and describe participant-reported obstetric, perinatal, and neonatal conditions among the first 200 eligible vaccine recipients enrolled after national introduction. The study was not designed to evaluate vaccine effectiveness, compare vaccinated and unvaccinated pregnancies, detect rare safety signals, or estimate causal effects.

An earlier version of this work was presented as an e-poster at VAC-LatAm 26 - Congreso Latinoamericano de Vacunología, held in Buenos Aires, Argentina, on June 24-25, 2026. A prior, non-identical version was posted to the Preprints.org preprint server on August 17, 2026 [11].

## Materials and methods

Study design and setting

We conducted a retrospective, descriptive, active-follow-up cohort study following STROBE (Strengthening the Reporting of Observational Studies in Epidemiology) recommendations [12]. Participants completed one structured interview after vaccination, generally after delivery; this was not prospective symptom surveillance. Vaccine administration was not a study procedure. Vaccinations occurred at public or private facilities in Panama from July through December 2025, and recruitment and interviews were conducted at CEVAXIN sites and, to a lesser extent, Vacunar Panamá from January through July 2026.

Participants

Potential participants were recruited by consecutive convenience sampling among women attending participating sites. Eligibility required age 18 years or older at vaccination, documented RSVpreF vaccination at 32.0-36.6 weeks of gestation, and availability of birth-weight information. Vaccination was verified from the participant's vaccination card. Counts of all women approached and those declining were not available, so the flow begins with completed questionnaires. The birth-weight requirement and recruitment generally after delivery preferentially selected pregnancies resulting in a reported birth and limited ascertainment of pregnancy losses, fetal deaths, and severe outcomes without an available birth weight. The protocol target of 200 was chosen to estimate frequent proportions with approximately ±5 percentage-point precision when the expected proportion was 15%; it was not powered for group comparisons or uncommon events.

Data collection and outcomes

Trained interviewers administered a structured questionnaire by telephone, virtual interview, or in person. Variables included maternal age, residence, gestational age at vaccination, vaccination subsector, local and systemic reactions reported for the first seven days, obstetric conditions reported after vaccination, birth outcomes, and neonatal conditions reported during the first 28 days. Exact vaccination dates and interview timestamps were not retained in the analytic dataset; therefore, the interval between vaccination and interview could not be reconstructed. The onset date and gestational week of obstetric conditions were not collected. Infant age at interview and actual follow-up duration were also unavailable, so questionnaire items on bronchiolitis and RSV infection during the first six months were not analyzed. Maternal and neonatal conditions were participant-reported and were not verified or adjudicated against clinical records.

The questionnaire did not collect parity, previous preterm birth, pre-existing hypertension, diabetes, body mass index or obesity, smoking, multiple gestation, or other baseline obstetric risk factors. Fever was recorded only as a participant-reported symptom; no measured temperature was collected, so it is reported as self-reported fever rather than objectively defined fever. Preterm birth was defined as birth before 37 weeks, low birth weight as less than 2,500 g, very low birth weight as less than 1,500 g, and macrosomia as 4,000 g or greater. The questionnaire term high blood pressure during pregnancy was grouped as a participant-reported hypertensive disorder. Pregnancy loss, fetal death after vaccination, and neonatal death during the first month were separate questionnaire items. Because no gestational-age threshold or clinical adjudication was available for pregnancy loss, the original questionnaire wording was retained. The instrument did not classify congenital conditions as major or minor.

Data management and statistical analysis

Eligibility and cleaning rules were applied programmatically. Continuous variables were summarized using the arithmetic mean and sample standard deviation (SD); categorical variables were summarized as counts and percentages using the number of non-missing observations for each variable as the denominator. For binary outcomes, two-sided 95% confidence intervals (CIs) for single proportions were calculated using the Wilson score method without continuity correction [13]. Denominators and missingness are reported where outcomes were not available for all participants. The study was descriptive, had no comparator group, and was not powered for between-group inference. Accordingly, no null-hypothesis significance tests, test statistics, p-values, or statistical significance thresholds were calculated or applied. No multivariable analyses were performed. Analyses used R version 4.6.1 (R Foundation for Statistical Computing, Vienna, Austria) [14] and components of the *tidyverse* [15].

Ethical considerations

The study followed the Declaration of Helsinki. The Panama Clinic Bioethics Committee for Research (CBI-TPC) approved the original protocol on December 9, 2025 (EC-CBITPC-238). During data collection from January through July 2026, nationwide recruitment and verbal consent documented in the study instrument preceded approval of version 3.0 on August 18, 2026. This temporal deviation was reported to CBI-TPC. In its signed determination of September 18, 2026 (CA-CBI-TPC-282; CBI-TPC-238-2025), the committee authorized the use of the data for final analysis and publication. The amendment did not alter the study objectives, design, product, safety outcomes, sample size, or study period. Participants received study information and provided verbal consent before the interview; participation was voluntary. No direct identifiers were collected, and data were coded and kept confidential.

Use of generative artificial intelligence

During preparation of the study protocol, manuscript, and editorial revision, the authors used Anthropic Claude (2025-2026 versions; Anthropic, San Francisco, CA) and OpenAI Codex (2026; OpenAI, San Francisco, CA) to assist with drafting, data-cleaning workflows, statistical-analysis code development, and editorial revision. All AI-assisted outputs, including the analysis code, reported statistics, statistical-methods wording, media legends, and manuscript text, were reviewed and verified against source data by the authors. Generative AI was not used to synthesize or alter image content; both figures were generated deterministically from the verified analytic dataset using the supplied R code. The authors edited the final text and code and take full responsibility for the accuracy and integrity of the work.

## Results

Among 225 completed questionnaires, 25 were excluded using mutually exclusive categories: vaccination outside 32.0-36.6 weeks only (n = 16), age younger than 18 years only (n = 7), both criteria (n = 1), and missing birth weight (n = 1). The analytic cohort comprised 200 participants. As birth-weight availability was required, the cohort represents pregnancies with a reported birth and cannot estimate the frequency of pregnancy loss or fetal death among all vaccinated pregnancies. Mean maternal age was 28.1 years (SD = 6.4); 66.0% lived in Panama province, 32.0% in Panama Oeste, and 74.0% were vaccinated in the public subsector (Table 1).

**Table 1:** Baseline characteristics of maternal respiratory syncytial virus vaccine recipients and their newborns (N = 200), Panama, 2025. Values are n (%) for categorical variables and mean ± SD for continuous variables. Percentages are based on N = 200. SD, standard deviation. No hypothesis tests were performed; therefore, test statistics, p-values, and a statistical significance threshold are not applicable.

Characteristic	n (%) or mean ± SD
Maternal age, years	28.1 ± 6.4
Maternal age: 18-24 years	68 (34.0)
Maternal age: 25-29 years	53 (26.5)
Maternal age: 30-34 years	40 (20.0)
Maternal age: 35 years or older	39 (19.5)
Gestational age at vaccination: 32 weeks	60 (30.0)
Gestational age at vaccination: 33 weeks	32 (16.0)
Gestational age at vaccination: 34 weeks	35 (17.5)
Gestational age at vaccination: 35 weeks	33 (16.5)
Gestational age at vaccination: 36 weeks	40 (20.0)
Province of residence: Panama	132 (66.0)
Province of residence: Panama Oeste	64 (32.0)
Province of residence: Other/outside/unspecified	4 (2.0)
Vaccination subsector: Public	148 (74.0)
Vaccination subsector: Private	40 (20.0)
Vaccination subsector: Unclassifiable	12 (6.0)
Gestational age at birth, weeks	38.9 ± 1.2
Birth weight, g	3,250 ± 447
Mode of delivery: Vaginal	130 (65.0)
Mode of delivery: Cesarean	70 (35.0)

Reactogenicity

At least one reaction during the first seven days was retrospectively reported by 111 participants (55.5%; 95% CI: 48.6-62.2). Local reactions (51.5%) were more frequent than systemic reactions (23.0%). Injection-site pain was the most frequent event (48.0%), followed by myalgia (13.5%), injection-site pruritus (9.0%), and induration (8.5%); self-reported fever occurred in 2.5% (Table 2 and Figure 1). Two participants reported persistence into the second week, one with local pain and one with fatigue. These frequencies are recall-dependent because the exact interview interval was unavailable.

**Table 2:** Retrospectively reported local and systemic reactions during the first seven days after maternal respiratory syncytial virus vaccination (N = 200). CI, two-sided 95% confidence interval calculated using the Wilson score method without continuity correction. Categories are not mutually exclusive. Symptoms were reported retrospectively in one interview; exact vaccination-to-interview intervals were unavailable. Fever was self-reported and was not based on a measured temperature. No hypothesis tests were performed.

Event	n (%)	95% CI
Injection-site pain	96 (48.0)	41.2-54.9
Injection-site pruritus	18 (9.0)	5.8-13.8
Induration	17 (8.5)	5.4-13.2
Erythema	9 (4.5)	2.4-8.3
Myalgia	27 (13.5)	9.5-18.9
Headache	15 (7.5)	4.6-12.0
Fatigue	14 (7.0)	4.2-11.4
Self-reported fever	5 (2.5)	1.1-5.7
Chills	4 (2.0)	0.8-5.0
Arthralgia	3 (1.5)	0.5-4.3
Nausea	1 (0.5)	0.1-2.8
Any local reaction	103 (51.5)	44.6-58.3
Any systemic reaction	46 (23.0)	17.7-29.3
Any reaction	111 (55.5)	48.6-62.2

**Figure 1:**
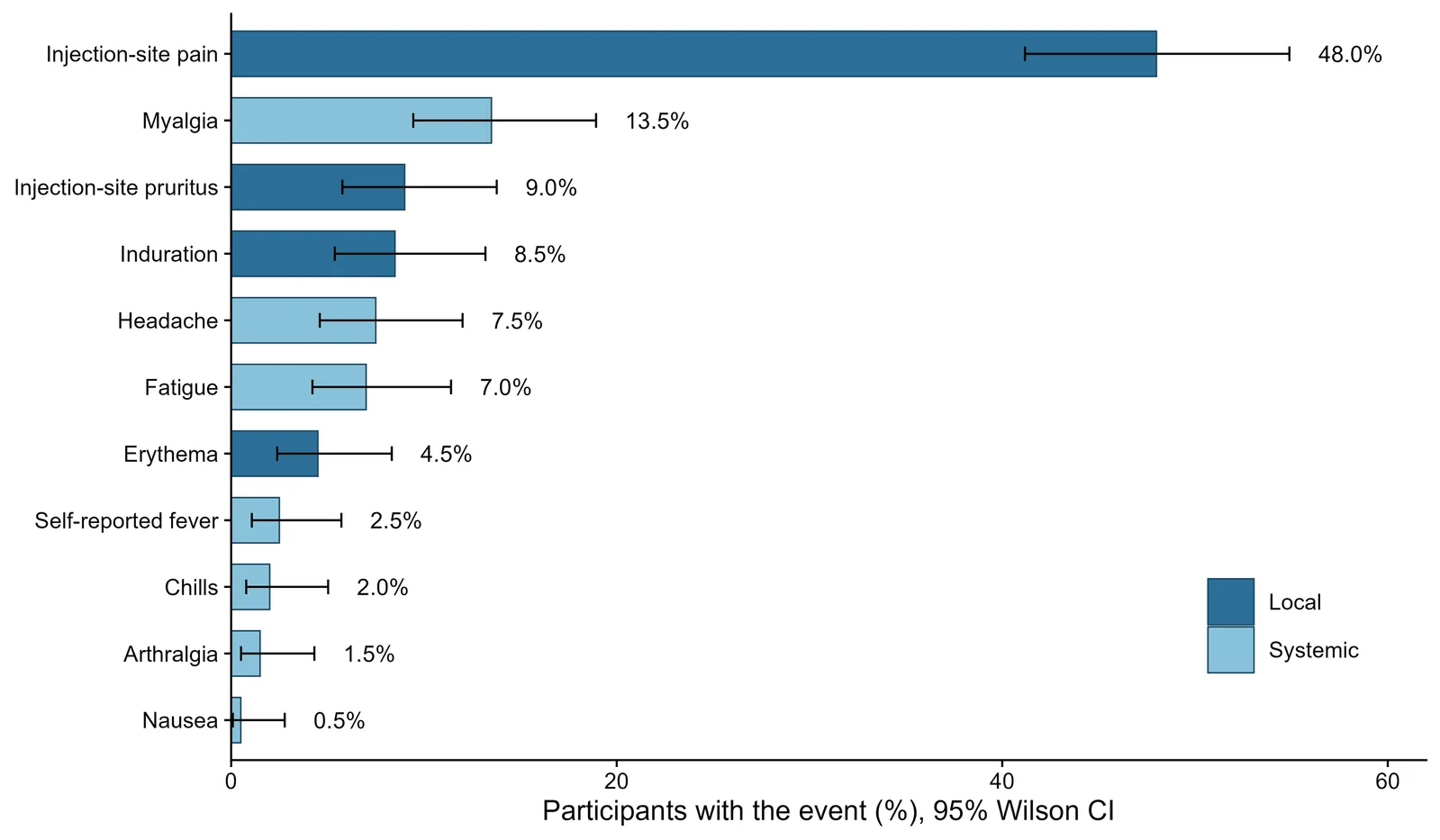
Frequency of retrospectively reported local and systemic reactions during the first seven days after maternal respiratory syncytial virus vaccination. Bars show percentages among 200 participants, and error bars show two-sided Wilson 95% confidence intervals without continuity correction. Categories are not mutually exclusive. Symptoms were reported in one retrospective interview; exact vaccination-to-interview intervals were unavailable. Fever was self-reported and not based on a measured temperature. No hypothesis tests were performed.

Obstetric conditions

Thirty-five participants (17.5%; 95% CI: 12.9-23.4) reported at least one obstetric condition. A participant-reported hypertensive disorder was the most frequent category (24; 12.0%), including gestational hypertension (18; 9.0%) and preeclampsia (11; 5.5%), with five participants reporting both labels. Preterm uterine contractions were reported by nine (4.5%) and obstetric hemorrhage by four (2.0%). No participant selected the questionnaire option pregnancy loss, and no thrombocytopenia, paralysis, facial paralysis, or Guillain-Barré syndrome was reported (Table 3). The onset date and gestational week of these conditions were not collected, so temporal proximity to vaccination cannot be established.

**Table 3:** Participant-reported obstetric conditions after maternal respiratory syncytial virus vaccination (N = 200). CI, two-sided 95% confidence interval calculated using the Wilson score method without continuity correction. Categories are not mutually exclusive. Diagnoses and labels were participant-reported and were not clinically verified. Five participants reported both gestational hypertension and preeclampsia. Onset dates and gestational weeks were unavailable. Pregnancy loss retains the questionnaire wording because no gestational-age definition was specified. No hypothesis tests were performed.

Event	n (%)	95% CI
Any participant-reported hypertensive disorder	24 (12.0)	8.2-17.2
Gestational hypertension	18 (9.0)	5.8-13.8
Preeclampsia	11 (5.5)	3.1-9.6
Preterm uterine contractions	9 (4.5)	2.4-8.3
Obstetric hemorrhage	4 (2.0)	0.8-5.0
Oligohydramnios	2 (1.0)	0.3-3.6
Clots or thrombi	2 (1.0)	0.3-3.6
Placental abruption	1 (0.5)	0.1-2.8
Premature rupture of membranes	1 (0.5)	0.1-2.8
Muscle weakness	1 (0.5)	0.1-2.8
Participant-reported pregnancy loss	0 (0.0)	0.0-1.9
Thrombocytopenia	0 (0.0)	0.0-1.9
Paralysis/facial paralysis	0 (0.0)	0.0-1.9
Guillain-Barre syndrome	0 (0.0)	0.0-1.9
Any participant-reported obstetric condition	35 (17.5)	12.9-23.4

Perinatal and neonatal outcomes

Mean gestational age at birth was 38.9 weeks (SD = 1.2) and mean birth weight was 3,250 g (SD = 447) (Figure 2). Four births were preterm (2.0%; 95% CI: 0.8-5.0); all late-preterm infants and five newborns had low birth weight (2.5%; 95% CI: 1.1-5.7). No participant reported fetal or neonatal death; however, post-delivery recruitment and the birth-weight requirement limited ascertainment of these outcomes, and the zero reports should not be interpreted as population frequencies or reassuring evidence. At least one neonatal condition was reported for 54 newborns (27.0%), most often jaundice (20.0%), followed by respiratory distress (4.0%) and neonatal sepsis (3.5%) (Table 4). Jaundice severity, bilirubin values, and phototherapy were not captured. Two participant-reported congenital conditions were described: one unspecified heart problem and one cleft lip/palate. Neither was clinically verified, adjudicated, or classified as major or minor. Apgar fields were missing for 22.5% at one minute and 23.0% at five minutes and were not clinically verified.

**Figure 2:**
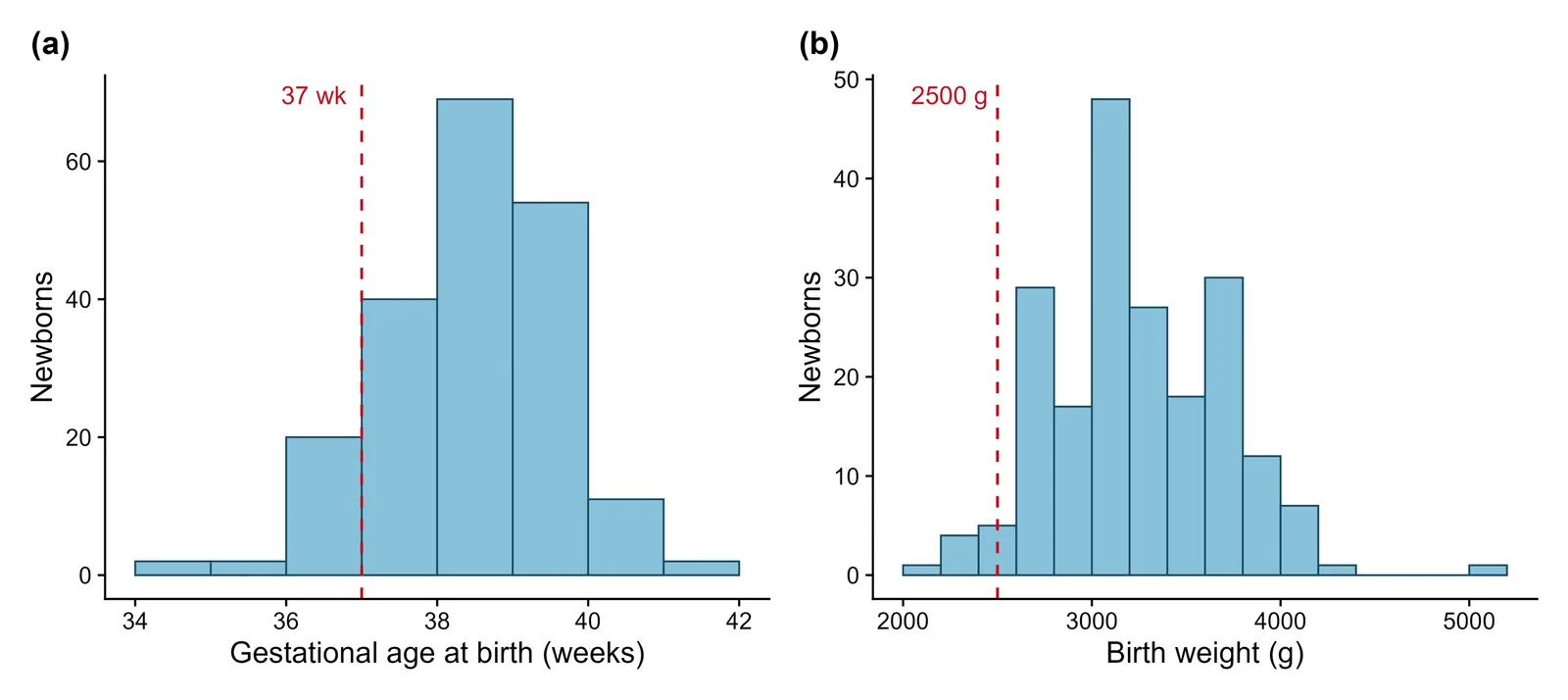
Distributions of gestational age at birth and birth weight among newborns of maternal respiratory syncytial virus vaccine recipients. Panel (a) shows gestational age at birth in weeks, and panel (b) shows birth weight in grams among the 200 included births. Dashed vertical lines indicate the thresholds for preterm birth at 37 weeks and low birth weight at 2,500 g. Because eligibility required vaccination beginning at 32 weeks and available birth weight, the distributions are descriptive of selected included births. No hypothesis tests were performed.

**Table 4:** Reported perinatal outcomes and early neonatal conditions among newborns of maternal respiratory syncytial virus vaccine recipients (N = 200). Percentages are based on N = 200. CI, two-sided 95% confidence interval calculated using the Wilson score method without continuity correction. Conditions were participant-reported and not clinically verified. The two congenital-condition reports were an unspecified heart problem and cleft lip/palate; neither was classified as major or minor. Post-delivery recruitment and the birth-weight requirement limited ascertainment of pregnancy loss and fetal death, so zero reports should not be interpreted as population frequencies or reassuring evidence. No hypothesis tests were performed.

Outcome	n (%)	95% CI
Fetal death after vaccination	0 (0.0)	0.0-1.9
Neonatal death (0-28 days)	0 (0.0)	0.0-1.9
Preterm birth (<37 weeks)	4 (2.0)	0.8-5.0
Very preterm birth (<32 weeks)	0 (0.0)	0.0-1.9
Low birth weight (<2,500 g)	5 (2.5)	1.1-5.7
Very low birth weight (<1,500 g)	0 (0.0)	0.0-1.9
Macrosomia (>=4,000 g)	10 (5.0)	2.7-9.0
Cesarean delivery	70 (35.0)	28.7-41.8
Participant-reported neonatal jaundice	40 (20.0)	15.1-26.1
Participant-reported respiratory distress	8 (4.0)	2.0-7.7
Participant-reported neonatal sepsis	7 (3.5)	1.7-7.1
Participant-reported neonatal hypoglycemia	3 (1.5)	0.5-4.3
Participant-reported congenital condition	2 (1.0)	0.3-3.6
Any participant-reported neonatal condition	54 (27.0)	21.3-33.5

## Discussion

This cohort provides an early description of retrospectively reported maternal and neonatal events during Panama's first months of routine RSVpreF vaccination. Reactogenicity was mainly local and short-lived, and four late preterm births and five low-birth-weight newborns were reported among the included births. These observations do not establish comparative safety, exclude rare events, or support causal attribution. Post-delivery recruitment and the birth-weight requirement also limit ascertainment of pregnancy loss and fetal death.

The study's novelty is geographic and programmatic rather than an evaluation of vaccine effectiveness. Panama is an early adopter in a tropical setting where RSV is detected year-round and seasonal delivery models may be less directly transferable [3,4]. The cohort includes vaccination in both public and private subsectors during the first national implementation period. The findings should be understood as an early Panamanian implementation description, not a baseline for Central America or other populations.

The retrospectively reported reactogenicity pattern was compatible with common reactions described in the pivotal trial, but event-specific frequencies are not directly comparable. Injection-site pain occurred in 48.0% here and 40.6% among RSVpreF recipients in MATISSE; self-reported fever occurred in 2.5% and protocol-defined fever in 2.6%, respectively [5]. MATISSE used prospective electronic diaries, measured definitions, and severity grading, whereas this study used one retrospective interview. Differences in recall interval, prompting, timing, measurement, and selection preclude direct comparison and may explain lower reporting of some systemic reactions.

Preterm birth remains a central safety outcome because of the numerical imbalance reported in MATISSE [10]. The four late preterm births in this cohort should be interpreted only as a descriptive count among included births. Vaccination eligibility beginning at 32 weeks creates left truncation because pregnancies ending earlier cannot enter the exposed cohort. Recruitment after delivery and the birth-weight requirement add survivor and outcome-dependent selection. Larger observational cohorts in Argentina and France found no increased adjusted risk of preterm birth or low birth weight after maternal RSVpreF vaccination [16,17], but this small, uncontrolled cohort cannot provide a comparative estimate.

Recent US studies add nuance. Sequential surveillance of 13,619 exposed pregnancies did not identify an increased preterm-birth risk, but residual confounding limited interpretation of possible elevations in hypertensive disorders and premature rupture of membranes [18]. A Vaccine Safety Datalink study of 13,966 vaccine recipients likewise found no increase in preterm birth, stillbirth, or small-for-gestational-age birth, while reporting a modest association with hypertensive disorders that requires further study [19]. The 24 participant-reported hypertensive disorders in this cohort cannot confirm or refute that signal because baseline obstetric risk factors were not collected, onset timing was unavailable, terminology was broad, and diagnoses were not clinically verified. This outcome should be prioritized for record-linked surveillance in Panama.

Strengths include vaccination-card verification, structured data collection, participation from both health subsectors, explicit analytic definitions, transparent reporting of denominators, and reusable analysis code. The study also demonstrates the operational feasibility of contacting vaccine recipients after routine maternal immunization. Its contribution is limited to common reported events and priorities for improved surveillance.

Limitations are substantial. Convenience recruitment through a small number of follow-up sites and the concentration of 98% of participants in Panama and Panama Oeste restrict generalizability. The number approached and refusal rate were unavailable. The exact vaccination-to-interview interval was not retained, so recall duration could not be quantified; retrospective self-report creates recall and classification error. Medical records were not reviewed, measured temperature was not collected, and most maternal and neonatal diagnoses were not clinically verified. Parity, previous preterm birth, pre-existing hypertension, diabetes, body mass index or obesity, smoking, multiple gestation, and other baseline obstetric risk factors were not collected. Onset dates or gestational weeks for obstetric conditions were unavailable. Infant age and follow-up duration were not retained, so six-month respiratory outcomes could not be analyzed. Eligibility beginning at 32 weeks, post-delivery recruitment, and the birth-weight requirement create left-truncation, survivor, and outcome-dependent selection. There was no contemporaneous comparator, adjustment for confounding, severity grading, formal causality assessment, or verified background rate. The sample cannot detect rare events or establish safety equivalence.

For program evaluation, national immunization records should be linked, under appropriate governance, to antenatal, delivery, and neonatal records; outcomes should be clinically verified; and contemporaneous comparators and prespecified confounder adjustment should be used. Multicenter recruitment beyond the metropolitan area, capture of all eligible pregnancies regardless of outcome, prospective documentation of interview timing, and multiyear accumulation would improve generalizability and precision. This staged approach aligns with WHO and PAHO emphasis on post-introduction monitoring and local evidence generation [6,7].

## Conclusions

During Panama's initial implementation period, retrospectively self-reported reactions among 200 maternal RSVpreF vaccine recipients were mainly local and short-lived. The reported maternal, perinatal, and neonatal conditions are descriptive; without a contemporaneous comparator or validated background rates, this study cannot determine whether any outcome occurred more often than expected, assess comparative safety, exclude rare signals, or support causal inference.

This cohort provides an early Panamanian implementation description and identifies priorities for stronger national surveillance. Clinical verification, record linkage, contemporaneous comparison groups, broader geographic coverage, inclusion of pregnancies regardless of outcome, and accumulated multiyear samples are needed.

## Appendices

English translation of the approved data-collection instrument, version 3.0

The original Spanish-language instrument dated July 29, 2026, approved by CBI-TPC on August 18, 2026, is the authoritative version and is supplied as a separate PDF. This English translation is provided for Cureus review.

Study Description and Voluntary Participation

The instrument explained that the observational study collected information from women vaccinated against respiratory syncytial virus during pregnancy in Panama, regardless of whether vaccination occurred at a public or private facility. It covered post-vaccination health events, gestational age at vaccination, pregnancy evolution, and newborn outcomes. Participation was voluntary. Respondents could skip any question or stop at any time without consequences. No direct identifiers were requested; data were coded and confidential. Submission of responses constituted voluntary acceptance to participate, and the platform recorded the completion date and time.

Section 1: Vaccination Center

1. Where were you vaccinated?

Required, single choice.

Response options: Ministry of Health facility; Social Security Fund facility; VACUNAR Panama/Pediatrics Clinic; Clinic at Hospital San Fernando; The Fertile Group; CEVAXIN; Another private hospital, clinic, or vaccination center; Do not remember.

Section 2. Demographic data

2. Country. Required, short text.

3. Province. Required, short text.

4. District. Required, short text.

5. Corregimiento. Required, short text.

If the district or corregimiento was unknown, respondents were asked to enter the area.

Section 3. Vaccination During Pregnancy

6. Age when the Abrysvo respiratory syncytial virus vaccine was received.

Required, numeric years.

7. Weeks of pregnancy when the vaccine was received.

Required, numeric weeks.

Questions 8-18 asked when each symptom occurred after vaccination. Each was required and used the same single-choice options: first week; second week; one and a half months; did not have this symptom.

8. Pain in the vaccinated arm.

9. Headache.

10. Muscle pain (myalgia).

11. Nausea.

12. Skin redness (erythema).

13. Skin induration at the injection site.

14. Itching (pruritus).

15. Fever.

16. Chills.

17. Joint pain without inflammation (arthralgia).

18. Fatigue.

19. Did you have another symptom? If so, which one?

Optional, open text.

20. Did you have any of the following problems after receiving the respiratory syncytial virus vaccine?

Required, multiple choice.

Response options: placental abruption; gestational hypertension; preeclampsia; oligohydramnios; blood clot or thrombosis; pregnancy loss; premature rupture of membranes; immune thrombocytopenia; thrombocytopenia; paralysis of any body part; facial paralysis; muscle weakness; Guillain-Barré syndrome; bleeding during pregnancy; uterine contractions during pregnancy; none of the above; other.

Section 4. Questions About the Baby

21. Gestational age at birth.

Required, numeric weeks.

22. Birth weight.

Required, numeric response.

23. Type of birth.

Required, single choice: vaginal birth; cesarean birth.

24. Apgar score below seven at one minute.

Required, single choice: yes; no; do not know.

25. Apgar score below seven at five minutes.

Required, single choice: yes; no; do not know.

26. Fetal death after vaccination.

Required, single choice: yes; no.

27. Death during the first month of life.

Required, single choice: yes; no.

28. Problems during the first month of life.

Required, multiple choice.

Response options: jaundice; hypoglycemia; sepsis; respiratory distress; congenital malformations; heart problems; neonatal hypokinesia; none of the above; other.

29. Bronchiolitis during the first six months of life.

Required, single choice: yes; no.

30. Respiratory syncytial virus infection during the first six months of life.

Required, single choice: yes; no.
