# Correction: Maternal Bivalent Respiratory Syncytial Virus Prefusion F (RSVpreF) Vaccination in Panama: A Retrospective Follow-Up Study

**DOI:** 10.7759/cureus.c482

**Published:** 2026-09-29

**Authors:** Ana Luca Quintero, Rodrigo DeAntonio, Isaac Fernandez Camargo, Ana Peralta Alvarado, Luis Escobar, Belen Ricci Perez, Luis Alberto Flores, Cynthia Gadea, Sofia Barrios, Emmanuel Guillen, Abigail De Avila, Jaime Ricardo Sevillano Giraldez

**Affiliations:** 1 Pediatrics, Vacunar Panama, Panama, PAN; 2 Department of Population Intelligence and Scientific Advancement, Endpoints Research Sites Network &amp; CEVAXIN, Panama, PAN; 3 Faculty of Medicine, Universidad de Panamá, Panama, PAN; 4 Pediatrics, Pediatrics Clinic Panama, Panama, PAN; 5 Population Intelligence and Biostatistics, Cevaxin, Panama, PAN; 6 Faculty of Medicine, School of Public Health, Universidad de Panamá, Panama, PAN

The affiliations for Rodrigo DeAntonio and Jaime Ricardo Sevillano Giraldez have been changed to: ‘Department of Population Intelligence and Scientific Advancement, Endpoints Research Sites Network & CEVAXIN, Panama, PAN’ and ‘Faculty of Medicine, School of Public Health, Universidad de Panamá, Panama, PAN’.

